# Adverse childhood experiences and prescription drug use in a cohort study of adult HMO patients

**DOI:** 10.1186/1471-2458-8-198

**Published:** 2008-06-04

**Authors:** Robert F Anda, David W Brown, Vincent J Felitti, Shanta R Dube, Wayne H Giles

**Affiliations:** 1 National Center for Chronic Disease Prevention and Health Promotion, Centers for Disease Control and Prevention, Atlanta, Georgia, USA; 2 Department of Preventive Medicine, Southern California Permanente Medical Group (Kaiser Permanente), San Diego, California, USA

## Abstract

**Background:**

Prescription drugs account for approximately 11% of national health expenditures. Prior research on adverse childhood experiences (ACEs), which include common forms of child maltreatment and related traumatic stressors, has linked them to numerous health problems. However, data about the relationship of these experiences to prescription drug use are scarce.

**Method:**

We used the ACE Score (an integer count of 8 different categories of ACEs) as a measure of cumulative exposure to traumatic stress during childhood. We prospectively assessed the relationship of the Score to prescription drug use in a cohort of 15,033 adult HMO patients (mean follow-up: 6.1 years) and assessed mediation of this relationship by documented ACE-related health and social problems.

**Results:**

Nearly 1.2 million prescriptions were recorded; prescriptions rates increased in a graded fashion as the ACE Score increased (p for trend < 0.0001). Compared to persons with an ACE Score of 0, persons with a Score ≥ 5 had rates increased by 40%; graded relationships were seen for all age groups (18–44, 45–64, and 65–89 years) (p for trend < 0.01). Graded relationships were observed for the risk of being in the upper decile of number of classes of drugs used; persons with scores of ≥ 5 had this risk increased 2-fold. Adjustment for ACE-related health problems reduced the strength of the associations by more than 60%.

**Conclusion:**

ACEs substantially increase the number of prescriptions and classes of drugs used for as long as 7 or 8 decades after their occurrence. The increases in prescription drug use were largely mediated by documented ACE-related health and social problems.

## Background

A growing body of literature suggests that child maltreatment and traumatic stressors have long-term consequences for adult health behavior and adult health outcomes. Adults with a history of child maltreatment have been shown to be at increased risk of multiple health risk behaviors and risk factors for chronic disease including overweight or obesity [1,2], smoking [3-5], and physical inactivity [6], and some have reported associations with higher prevalences of heart [7-9], lung [10], and liver[11] disease, diabetes [9], and depression or depressive disorders [12-17] – chronic conditions associated with higher health care service utilization.

Health care resources are scarce, and therefore research focused on explaining and/or predicting utilization of health care services is both of interest and of importance to policymakers. Use of prescription drugs, one of the most rapidly growing sectors of health care in the US, has increased substantially over the past 10 to 15 years, although use of some prescription drugs, such as hormone replacement therapy [18] and alpha- (α-) blockers [19], has decreased. In 2000, three in five US adults filled at least one prescription. [20] Nationally, the number of purchased prescription drugs increased 75% between 1993 and 2005 from 2.0 to 3.5 billion per year. [21,22] In 2004 an estimated 45% of American adults were taking prescription drugs on a regular basis and 27% were taking them occasionally [23].

Increases in prescribed drug use are reflected in expenditures for these medications. Spending on prescription drugs in the United States in 2003 was nearly $180 billion, or 11% of total national health expenditures, and was more than four times the amount spent on prescription drugs in 1990.[24] Future spending on prescription drugs is projected to increase 10.5% between 2005 and 2013, but this may turn out to be an underestimate as the projection was made before the passage of the Medicare Prescription Drug, Improvement, and Modernization Act of 2003 [25].

Few studies have assessed the relationship of child maltreatment and related traumatic stressors to the use of prescription drugs in adulthood. [26-29] Our earlier analyses of data from the Adverse Childhood Experiences (ACE) Study have demonstrated that an integer count of the number of categories of abuse, exposure to domestic violence, and other forms of serious household dysfunction (the ACE Score) [30] experienced during childhood has a strong graded relationship to a wide variety of health and social problems from adolescence to adulthood (Table 1). [2,3,6,7,11,12,31-43] Therefore, we hypothesized that the ACE Score would be associated with increased rates and number of classes of prescriptions drugs used during an average of 6.1 years of follow-up of adults in the ACE Study cohort.

**Table 1:** Health and social problems shown to have a graded relationship to the ACE Score

**Type of Problem**	**Outcomes Associated with Adverse Childhood Experiences**
Prevalent Diseases	Ischemic heart disease^6,7 ^cancer,^6 ^chronic lung disease,^6 ^skeletal fractures,^6 ^sexually transmitted diseases,^6,32,33 ^and liver disease^6,11^
Risk Factors for Common Diseases/Poor Health	Smoking^,3,6,31,32 ^alcohol abuse,^12,31,32,34 ^promiscuity,^6,31,32,35 ^obesity,^2,6,31 ^illicit drug use,^6,31,32,36 ^injected drug use,^6,31,36 ^multiple somatic symptoms,^31 ^poor self-rated health,^6 ^high perceived risk of AIDS^35^
Poor Mental Health	Depressive disorders,^6,31,32,37 ^anxiety,^31 ^hallucinations,^31,38 ^panic reactions,^31 ^sleep disturbances,^31 ^memory disturbances,^31,39 ^poor anger control,^31 ^risk of perpetrating or being a victim of domestic violence^31^
Sexual and Reproductive Health	Early age at first intercourse,^31,35 ^sexual dissatisfaction,^31 ^teen pregnancy,^40 ^unintended pregnancy,^41 ^teen paternity,^42 ^fetal death^40^
General Health and Social Problems	High perceived stress,^31 ^difficulty with job performance,^43 ^relationship problems,^43 ^marriage to an alcoholic^34^

* A complete bibliography of ACE Study publications listed by topic area is available.^57^

## Methods

### Study population

The ACE Study methods have been described in detail elsewhere. [3,6,31] Briefly, more than 50,000 adult members of the Kaiser Health Plan in San Diego, California are evaluated annually for wellness care at Kaiser Permanente's San Diego Health Appraisal Clinic (HAC) which includes a standardized health history, psychosocial evaluations, and physical examination. Appointments for most members are obtained by self-referral with 20% referred by their health care provider. [6] A review of continuously enrolled (between 1992 and 1995) Kaiser Permanente members aged 25 years or older in San Diego revealed that 81% of those members had been evaluated at the Health Appraisal Clinic. [6] The ACE Study was approved by the institutional review board of Kaiser Permanente and written informed consent was given by study participants.

Within two weeks of their HAC visit, each member who completed the standardized evaluation during the baseline survey period (August 1995 to October 1997) was mailed a study questionnaire that contained questions about childhood exposure to abuse, neglect, domestic violence, and other forms of serious and interrelated household dysfunction. [30] A total of 17 421 (68%) responded; 84 persons had incomplete information on race and educational attainment leaving an analytic sample of 17 337 persons [32].

### Eligibility for the Prospective (Follow-up) Phase of the Study

Follow-up data on the use of prescription drugs was available from Kaiser Permanente from January 1, 1997 through December 31, 2004. We excluded 18 persons aged 90 years or older to facilitate direct age standardization to the 2000 US population. We also excluded 1053 (6%) persons whose membership had lapsed prior to their evaluation at the HAC or before January 1, 1997 or whose member record number was not considered valid.

In addition, among persons who disenrolled and reenrolled at least once (median/mean: 1 time; range: 1–6 times) during the follow-up period, we excluded the 1233 (7%) persons whose ratio of time disenrolled/total possible time enrolled during follow-up exceeded 20%; we considered such persons to have inadequate continuity of follow-up to merit inclusion in the prospective analysis. Thus, the final sample retained 15 033 of the 17 337 persons included in the baseline survey (87%).

To assess any influence of including persons with discontinuous follow-up who had a ratio as described of (≤ 20%), we repeated the analyses included herein after excluding any persons whose follow-up was discontinuous.

### Relationship of the ACE Score to Exclusion from Follow-up

We used logistic regression to assess the probability that the ACE Score might be related to excluding from follow-up. In this analysis, the risks (ORs) of exclusion from follow-up for persons with 1, 2, 3, or ≥ 4 ACEs were 1.0 (0.9–1.1), 1.0 (0.9–1.2), 1.2 (1.0–1.4) and 1.1 (0.9–1.3), respectively, and thus we ruled out exclusion from follow-up as a potential source of bias.

### Definitions of Adverse Childhood Experiences (ACEs)

All questions used to define ACEs pertained to the respondents' first 18 years of life (≤ 18 years of age) (Table 2). Questions adapted from the Conflict Tactics Scale (CTS) [44] had 5 response categories: "never", "once or twice", "sometimes", "often", or "very often". We defined 3 types of childhood abuse: emotional abuse (2 questions), physical abuse (2 questions), or contact sexual abuse (4 questions) by Wyatt. [45] We also defined 5 exposures to household dysfunction during childhood: exposure to substance abuse (defined by 2 questions) [46], mental illness (2 questions), violent treatment of mother or stepmother (4 questions) [44], criminal behavior in the household (1 question), and parental separation or divorce (1 question). Respondents were defined as exposed to a category if they responded "yes" to 1 or more of the questions in that category.

**Table 2:** Definition and prevalence of each category of adverse childhood experience

**Childhood Abuse**	
*Emotional*	10.3%
(Did a parent or other adult in the household.....) 1) Often or very often swear at you, insult you, or put you down? 2) Sometimes, often, or very often act in a way that made you that you might be physically hurt?	
	
**Physical**	28.0%
(Did a parent or other adult in the household.....) 1) Often or very often push, grab, slap, or throw something at you? 2) Often or very often hit you so hard that you had marks or were injured?	
	
**Sexual**	20.4%
(Did an adult or person at least 5 years older ever.....) 1) Touch or fondle you in a sexual way? 2) Have you touch their body in a sexual way? 3) Attempt oral, anal, or vaginal intercourse with you? 4) Actually have oral, anal, or vaginal intercourse with you?	
	
Household dysfunction	
**Substance abuse**	26.6%
1) Live with anyone who was a problem drinker or alcoholic? 2) Live with anyone who used street drugs?	
	
**Mental Illness**	19.0%
1) Was a household member depressed or mentally ill? 2) Did a household member attempt suicide?	
	
**Mother treated violently**	12.6%
(Was your mother (or stepmother)): 1) Sometimes, often, or very often pushed, grabbed, slapped, or had something thrown at her? 2) Sometimes, often, or very often kicked, bitten, hit with a fist, or hit with something hard? 3) Ever repeatedly hit over at least a few minutes? 4) Ever threatened with or hurt by a knife or gun?	
	
**Incarcerated household member**	4.5%
1) Did a household member go to prison?	
	
**Parental separation or divorce**	22.8%
1) Were your parents ever separated or divorced?	
	
**Number of adverse childhood experiences (ACE Score)**	
0	36.4%
1	26.2%
2	15.9%
3	9.3%
4	6.1%
5 or more	6.1%

* Percentages based on a total of 15,033 adults included in the analysis sample

To assess the cumulative effect of early trauma and stress on use of prescription drugs, the total number of these categories of childhood exposures (range: 0–8) was summed to create the ACE Score (Table 2). The statistical characteristics and validity of the ACE Score have been published elsewhere [30].

### Rates of Prescription Drug Use

To calculate rates of prescription drug use, we divided total claims for prescription drugs by the cumulative person-time at risk during follow-up. Person-time at risk was calculated using electronic enrollment data files provided by Kaiser Permanente. Because pharmacy claims data were not available before January 1, 1997, we designated this date as beginning of follow-up for persons enrolled before January 1, 1997. We counted prescription medications through December 31, 2004 (latest available data).

We calculated the maximum possible person-time at risk as the difference between December 31, 2004 and the later of either January 1, 1997 or the baseline appointment date for persons who were continuously enrolled. For persons with periods of disenrollment from their baseline appointment date to December 31, 2004, follow-up time was calculated as the maximum possible person-time less periods of disenrollment.

### Use of Multiple Classes of Prescription Drugs

The Kaiser pharmacy database includes 15 classes of drugs: anti-infectives, endocrine and metabolic, cardiovascular, respiratory, gastrointestinal, genitourinary, central nervous system, analgesics and anesthetics, neuromuscular, topical products, biologics, antineoplastics, nutritional, hematological agents, and other drugs. On an *a priori *basis we chose the approximate age-specific upper decile of the number of classes of prescription drugs used during follow-up to define persons as having used multiple classes of pharmaceuticals. The approximate upper deciles for the age groups 18–44 years, 45–64 years, and 65–89 years were 8, 10, and 11, respectively; the prevalence of persons in each of these age-specific groups was 17%, 12%, and 15%, respectively (total = 14%).

### Statistical Analysis

Analyses were completed using SAS v8.2 (SAS Institute, Cary, NC). [47] We used the 2000 US Standard Population for direct age-standardization of prevalences and risks.

To assess the independent relationship between the ACE Score and the rate of prescription drug use, we used rate ratios derived from multivariable-adjusted negative binomial regression models employing PROC GENMOD. [47] To allow for differing lengths of follow-up, the log of person-time was incorporated as the offset in the model. Rate ratios were obtained by exponentiating estimated regression coefficients. Age, race/ethnicity, educational attainment and sex were forced into all models.

We used multivariable adjusted logistic regression to assess the relationship of the ACE Score to prescription of multiple classes of drugs and included the following covariates: age at baseline, sex, race (white, nonwhite), and education (< high school, high school, some college, college graduate). All statistical inferences were based on a significance level of α (2-sided) = 0.05.

### Assessment of Mediation by Documented ACE-related Health and Social Problems

Previously, we have reported graded relationships between the ACE Score and multiple health and social outcomes (Table 1) that ultimately lead a physician to prescribe medication. Therefore, we hypothesized that the presence of these conditions would mediate the relationship of the ACE Score to use of prescription pharmaceuticals. To examine this issue, we used logistic regression models without (Model A) and with (Model B) each of the health and social problems from Table 1 (coded dichotomously (yes/no). We compared the reduction in the strength of the relationship (odds ratio) between Model A and Model B using the risk decrement which was expressed as a percent and calculated as follows:

$$\frac{\text{Model A odds ratio}-\text{Model B odds ratio}}{\text{Model A odds ratio}-\text{1 }}\times 100.$$

## Results

### Characteristics of Study Population

The study population included 8134 women (54%) and 6899 men (46%). The mean age (standard deviation) was 57 (15) years. Seventy-six percent were white, 11% Hispanic, 4% Black, 7% Asian, < 1% Native American, and 2% other; 40% were college graduates; 36% had some college education; and 17% were high school graduates. Only 7% had not graduated from high school. The mean length of follow-up was 6.1 (SD, 2.4) years.

The prevalence of each of the 8 individual ACEs and the ACE Score are presented in Table 2. A total of 1 188 052 prescriptions were written during 95 883 person-years of follow-up. The age-standardized prescription rate was 9.6 per person-year (data not shown).

### Prescription Rates by ACE Score

As the ACE Score increased, rates of prescription drug use increased in a graded fashion (Table 3). This relationship was graded for each age group but was attenuated among persons 65–89 years. However, the trend was statistically significant for all age groups (Table 3).

**Table 3:** Prescription drug rates (per person per year) by age group and ACE Score

	Age 18–44 years		Age 45–64 years		Age 65–89 years	
ACE Score	Rate	RR* (95% CI)	Rate	RR* (95% CI)	Rate	RR* (95% CI)
0	5.03	1.00 (referent)	10.18	1.00 (referent)	17.27	1.00 (referent)
1	5.99	1.14 (1.00–1.29)	11.19	1.09 (1.02–1.17)	16.92	0.99 (0.94–1.05)
2	6.86	1.30 (1.13–1.50)	11.33	1.12 (1.04–1.21)	18.10	1.08 (1.00–1.16)
3	6.95	1.33 (1.13–1.56)	12.11	1.17 (1.06–1.28)	17.83	1.04 (0.93–1.15)
4	7.64	1.46 (1.23–1.73)	12.59	1.24 (1.11–1.39)	19.43	1.12 (0.98–1.29)
5 or more	8.84	1.63 (1.38–1.93)	15.48	1.45 (1.30–1.62)	20.67	1.25 (1.05–1.48)
Overall	6.45		11.32		17.47	
p-value, trend		< 0.0001		< 0.0001		0.0025

* Relative rate (RR) and 95% confidence interval (CI) adjusted for age, sex, race, education

### Risk of Being Prescribed Multiple Classes of Drugs

As the ACE Score increased, the risk (adjusted odds ratio) of having been prescribed multiple classes of pharmaceuticals also increased in a graded fashion for all age groups (Table 4).

**Table 4:** Prevalence of multiple prescription medication classes (upper decile by age) during follow-up by age group and ACE Score

	Age 18–44 years		Age 45–64 years		Age 65–89 years	
ACE Score	Top 10% (%)	OR* (95% CI)	Top 10% (%)	OR* (95% CI)	Top 10% (%)	OR* (95% CI)
0	12.67	1.00 (referent)	10.14	1.00 (referent)	14.06	1.00 (referent)
1	16.27	1.39 (1.06–1.82)	11.51	1.14 (0.93–1.41)	15.14	1.14 (0.94–1.39)
2	17.54	1.53 (1.14–2.04)	12.08	1.21 (0.96–1.53)	14.56	1.11 (0.87–1.43)
3	18.42	1.51 (1.09–2.09)	13.44	1.38 (1.05–1.80)	16.72	1.26 (0.90–1.76)
4	18.48	1.47 (1.04–2.07)	16.38	1.65 (1.22–2.24)	22.42	1.83 (1.24–2.72)
5 or more	25.27	2.11 (1.54–2.89)	21.52	2.22 (1.68–2.92)	19.23	1.56 (0.93–2.60)
Overall	16.97		12.31		14.98	
p-value, trend		< 0.0001		< 0.0001		0.0020

* Odds ratio (OR) and 95% confidence interval (CI) adjusted for age, sex, race, education

### Relationship Between Prescription Rates and Multiple Classes Drugs Used

As the number of classes of drugs used increased, the number of prescriptions also increased (Pearsons correlation = .69; p < 0.001). Both the mean and the median rates of prescriptions increased dramatically as the number of classes increased from 0 to 14 (data not shown).

### Mediation by ACE-related Health and Social Problems

For persons of all ages, the ACE Score had a graded relationship to rates of prescription drug use (Table 5); for persons with ACE Scores ≥ 5, the rate increased by more than 40% (Table 5). When we entered the variables for the health and social problems (Table 1) into a model for rates of prescriptions, the relative rates were reduced for each level of the ACE Score; these reductions ranged from 67–70% (median, 69%)(Table 5).

**Table 5:** Rates of prescription drugs and risk (odds ratio) of multiple classes prescribed by ACE Score with and without adjustment for potential mediating health-related conditions shown to have a graded relationship to the ACE Score

	Prescription drug rates			
	
ACE Score	Rate PPPY*	Model A RR† (95% CI)	Model B RR‡ (95% CI)	% Decrease due to mediation
0	8.41	1.00 (referent)	1.00 (referent)	···
1	9.40	1.06 (1.01–1.11)	1.02 (0.98–1.07)	67
2	9.96	1.13 (1.07–1.19)	1.04 (0.99–1.10)	69
3	10.28	1.15 (1.08–1.23)	1.05 (0.99–1.12)	67
4	11.58	1.25 (1.16–1.35)	1.06 (0.98–1.15)	76
5 or more	12.05	1.44 (1.33–1.56)	1.13 (1.05–1.23)	70
Overall	9.59			
p-value, trend		< 0.0001	0.0016	Median = 69

	Multiple prescription medication classes			
	
ACE Score	Proportion in Top Decile*	Model A OR† (95% CI)	Model B OR‡ (95% CI)	% Decrease due to mediation

0	10.84	1.00 (referent)	1.00 (referent)	···
1	13.49	1.19 (1.05–1.35)	1.14 (1.00–1.29)	26
2	14.19	1.24 (1.08–1.43)	1.08 (0.93–1.26)	67
3	15.70	1.33 (1.13–1.58)	1.11 (0.93–1.33)	67
4	17.76	1.57 (1.30–1.90)	1.22 (1.00–1.50)	61
5 or more	20.71	2.00 (1.67–2.40)	1.41 (1.15–1.73)	59
Overall	14.05			
p-value, trend		< 0.0001	0.0026	Median = 61

PPPY, per person per year

* Age standardized using the method of direct standardization and the 2000 US standard population

† Adjusted for age, sex, race, education

‡ Adjusted for age, sex, race, education, and all documented health-related problems shown to have a graded relationship to the ACE Score (see Table 1)

Similarly, for persons of all ages, the risk of being prescribed multiple classes drugs increased in a graded fashion as the ACE Score increased (Model A, Table 5); for persons with ACE Scores s805; 5, the risk was increased 2-fold. Entry of the ACE-related health problems into this model reduced the risks (ORs) by 26–67% (median: 61%).

## Discussion

In this large study of HMO patients we found that rates of prescriptions increased in a graded fashion as the ACE Score increased. This pattern was particularly evident in the younger age groups – whose rates were increased by as much as 60% for persons with ACE Scores of 5 or more. Previous research has shown ACEs to be associated with earlier onsets of health risks such as smoking [3], alcohol [34] and illicit drug use [36], and sexual intercourse. [31,35] Thus, ACEs may "accelerate" the onset of health risks and illnesses, in the process increasing the use of prescription drugs among younger persons. If this is the case, the greatest relative effects on prescription rates would be expected among younger persons.

Among older persons, the graded relationship between the ACE Score and prescription rates was attenuated. This could be due to differential morbidity and mortality because ACEs influence a multitude of health and social problems (Table 1). Older persons affected by ACEs might expectedly have higher levels of multimorbidity [31] or severe health problems (such as ischemic heart disease [7] or liver disease [11]), requiring ongoing specialty care. In this scenario, older persons would be less likely to visit a clinic for wellness care and hence, less likely to have enrolled in the study. Moreover, ACE Scores tend to be lower among older persons, [6] possibly as a result of increased mortality over time leading to a decreased likelihood that persons with high levels of ACEs would survive to be in the older age groups included in the study.

Our finding that the risk of being prescribed multiple classes of drugs during follow-up increased in a strong graded fashion as the ACE Score increased lends further support for the idea that ACEs matter long after they occur. We found a 2-fold increase for young and middle-aged persons and 1.7-fold for the older persons with Scores ≥ 5. Thus, there was little evidence of attenuation among older persons as was observed for rates of prescription use. This is likely due to the choice of the upper decile of number of drug classes – an extreme measure – which may have selected persons with comorbid conditions that resulted from exposure to ACEs. We have previously shown that the mean number of a variety of health-related problems [31] and the number of risk factors for the leading causes of death increases as the ACE Score increases [6].

When we controlled for documented ACE-related health and social problems (Table 1), the apparent effects of ACEs on rates of prescription drug use were reduced by 67–76% (median, (69%); similarly, the risk of using a high number of classes of drugs during follow-up was reduced by 26–67% (median, 61%). Thus, as would be expected, the documented ACE-related conditions among participants appear to account for the majority, although not all, of the increase use of prescription medications we observed. Because screening for childhood traumatic stressors is not yet a routine part of adult medical care, some clinicians are likely identifying and treating these conditions without a full understanding of their origins in the long-term neurobiologic effects [31,48,49] of childhood stressors.

Relationships between child maltreatment and prescription drug use in adulthood among adult survivors of child maltreatment have been examined previously, but the relationships remain unclear as studies are often limited by study design, use of clinical populations versus community-based samples, self-reported health care service utilization measures, examination of only one or two types of maltreatment, and suboptimal statistical analyses as in the case of no multivariable adjustment of comparisons. In our study, the multivariable-adjusted relative rate of prescriptions as well as the relative risk of use of a high number of classes of drugs increased with a higher ACE Score. In a study of 3333 women aged 18–64 years who were members of a large health maintenance organization in the northwestern United States, Bonomi and collegues [26] observed that women with a history of both physical and sexual childhood abuse had more pharmacy fills (adjusted incidence rate ratio = 1.57; 95% CI: 1.33–1.86) than women without a history of child physical or sexual abuse. In a study of 150 women aged 17–49 years seen consecutively for non-emergency medical care by a family practitioner in a health maintenance organization, Sansone and colleagues [29] found a significant association between sexual abuse and the number of prescribed medications, obtained from a physician review of patient medical records, during the 12-months following completion of a clinic survey; lifetime physical or emotional abuse were not associated with use. Participants were not queried for abuse that occurred only during childhood; rather, for each type of abuse participants provided an age range during which the event(s) occurred.

Using data from women members of a health maintenance organization, Farley and Patsalides [27] report significantly more prescription medications obtained from medical record review among women with a history of childhood physical and sexual abuse (*n *= 27) compared to women without a history of either form of abuse (*n *= 26). The study is limited by a low response rate (14%) for the mailed survey, the absence of any quantitative data on the prescription drug use, and the absence of any multivariable adjustment in the statistical comparisons of abuse groups. In a clinical sample of 75 women with fibromyalgia, Alexander and associates [28] observed an increased use of pain medications and greater outpatient service use among women with a history of sexual or physical abuse compared to those without such a history. The study did not stipulate when the abuse occurred (i.e., childhood or adolescence vs adulthood) and did not include multivariable-adjusted statistical analyses.

The analyses herein have several strengths. Prescriptions were obtained prospectively from electronic, administrative pharmacy claims data and therefore are not subject to differential misreporting. The relationship of ACEs to prescription drug use is not limited to any specific class of drugs (data not shown). Future analyses will detail the relationship of ACEs to increased use of individual classes of drugs.

Our results should be interpreted keeping the following limitations in mind. Because of the sensitive nature of questions about ACEs and affective problems, the responses probably represent an underreporting of their actual occurrence. However, our estimates of the prevalence of childhood exposures are similar to estimates from nationally representative surveys [50,51] indicating that the experiences of our participants are comparable to those of the larger population of adults. For example, in our study we found that 16% of the men and 25% of the women met the case definition for contact sexual abuse; a national telephone survey of adults conducted by Finkelhor and colleagues [52] using similar criteria for sexual abuse estimated that 16% of men and 27% of women had been sexually abused. Of the men in our study, 28% had been physically abused as boys, which closely parallels the percentage (31%) found in a population-based study of men in Ontario that used questions from the same scales. [53] The similarity in estimates of the prevalence of these childhood exposures between the ACE Study and other population-based studies suggests that our findings are likely to be applicable in other settings. Also, when utilizing retrospective reports of adverse childhood experiences, several factors may inevitably lead to variability in the responses over time. These include difficulty recalling the experiences the due to the time lapse between the events in question and the research survey, variability in responses may occur due to the sensitive nature of the questions and the subjects' knowledge of the "social taboos" of responding to such questions, and incomplete or total inability to recall the experiences due to memory impairments as a result of stressful childhood experiences. Dube and associates [54] observed that retrospective responses to childhood abuse and related forms of serious household dysfunction are generally stable over time concluding that there is good to excellent reliability in the reports of adverse childhood experiences during adulthood.

## Conclusion

The mind-body dichotomy that persists in Western medical training may lead clinicians away from understanding the role that childhood trauma and stress has on the health of their adult patients. Childhood stressors are known to produce changes in the developing brain that affect emotions, behavior, and cognition [55] which in turn can impair health and quality of life via numerous pathways (Table 1). These traumatic pathophysiological insults may be "silent" until much later in life, [55,56] leading clinicians to prescribe medications to treat symptoms and illnesses without knowledge of their potential origins in the disruptive effects of ACEs on neurodevelopment. As improvements in the treatment of persons affected by traumatic stress evolve, understanding the role of these childhood experiences on adult health will become increasingly important in making decisions about prognosis, diagnosis, and treatment.

## Competing interests

The authors declare that they have no competing interests.

## Authors' contributions

Study conception and design: RFA, DWB. Acquisition of data: RFA, VJF, SRD. Analysis and interpretation of data: RFA, DWB, WHG. Drafting of manuscript: RFA, DWB, VJF, SRD, WHG. Critical revision: RFA, DWB, VJF, SRD, WHG. All authors read and approved the final manuscript.

## Pre-publication history

The pre-publication history for this paper can be accessed here:


